# Case Report: Cardiac Involvement by Lymphoma: Rare but Heterogeneous Condition With Challenging Behaviors

**DOI:** 10.3389/fonc.2021.665736

**Published:** 2021-04-27

**Authors:** Elisa Lucchini, Marco Merlo, Mario Ballerini, Aldostefano Porcari, Gianfranco Sinagra, Lorenzo Pagnan, Marco Rensi, Andrea Romano, Rossana Bussani, Laura Ballotta, Francesco Zaja

**Affiliations:** ^1^ S.C. Ematologia, Azienda Sanitaria Universitaria Giuliano Isontina, Trieste, Italy; ^2^ S.C. Cardiologia, Dipartimento Cardiotoracovascolare, Centro per la Diagnosi e Cura delle Cardiomiopatie, Azienda Sanitaria Universitaria Giuliano Isontina, Trieste, Italy; ^3^ Dipartimento di Scienze Mediche e Chirurgiche e della Salute (DSM), Università degli Studi, Trieste, Italy; ^4^ S.C. Radiologia diagnostica ed Interventistica, Azienda Sanitaria Universitaria Giuliano Isontina, Trieste, Italy; ^5^ S.C. Medicina Nucleare, Azienda Sanitaria Universitaria Friuli Centrale, Udine, Italy; ^6^ S.C. (UCO) Anatomia ed Istologia Patologica, Azienda Sanitaria Universitaria Giuliano Isontina, Trieste, Italy

**Keywords:** lymphoma, diffuse large B cell lymphoma, marginal zone lymphoma, MRI, PET/CT (18)F-FDG, central nervous system relapse/progression, heart involvement, primary cardiac lymphoma

## Abstract

Cardiac lymphomas are rare extranodal lymphomas involving primarily and secondarily the heart and/or pericardium. Here we describe three cases of cardiac involvement from lymphoma with specific peculiarities: two primary cardiac Diffuse Large B-cell Lymphomas and one secondary involvement from Marginal Zone Lymphoma (MZL). The first case highlights the issue of early CNS relapse and the possible role for CNS prophylaxis; the second case demonstrates the difficulties of interpretation and possible mistakes of different radiologic techniques adopted to evaluate cardiac involvement by lymphoma during follow-up; the third is a unique case of MZL with cardiac involvement. Our aim is to share the findings observed in these cases putting them in relation with data from the literature.

## Introduction

Primary cardiac lymphoma (PCL), an extranodal lymphoma involving only the heart and/or pericardium, is a rare entity, accounting for 2% of primary cardiac tumors and 0.5% of extranodal lymphomas (1). It is more frequent in males, with a median age around 63 years, even if it can occur at any age (2). PCL involves more frequently the right side of the heart, right atrium being the most frequently affected site, followed by right ventricle, left ventricle, left atrium, atrial septum and ventricular septum (3). Due to its peculiar location, PCL clinically manifests with cardiac symptoms, usually secondary to congestive heart failure (54-64%) (2). Chest pain and rhythm alterations are also common presenting symptoms (2, 4), the latter manifesting as atrial arrhythmias or atrioventricular block, less frequently left or right bundle branch block and ventricular arrhythmias; rarely, sudden death can be the initial presentation of PCL. Pericardial effusion is frequently found (up to 58% of patients) (2), which may be complicated with cardiac tamponade. Due to its aggressive histology, together with cardiac symptoms, constitutional and “B” symptoms can also be found (4).

PCL is more often an aggressive lymphoma, diffuse large B cell lymphoma (DLBCL) being the most common histology (2–5); other histologies have been more rarely reported (6). Diagnosis of PCL requires histologic sample, which can be obtained by endomyocardial biopsy or even by surgical resection, when debulking is clinically needed (7). In selected cases, cytological examination of pericardial fluid may allow to omit a biopsy approach, if monoclonal lymphocytes are found by flow cytometry analysis. Echocardiography is in the majority of cases the first imaging evaluation, allowing a quick assessment of the local extent of the disease (3). Cardiac magnetic resonance imaging (MRI) is the gold standard imaging technique for cardiac masses, commonly hypointense on T1-weighted and hyperintense on T2-weighted sequences (8, 9). CT-scan and 18-FDG-PET/CT are useful to assess the extension of the disease, thus confirming the diagnosis of PCL. There are no guidelines for the management of PCL. Chemo-immunotherapy regimens used in aggressive B-cell lymphomas, such as R-CHOP or R-CHOP like regimens, are the standard first-line choices in DLBCL; chemotherapy regimens then vary according to the specific histology. PCL has historically been considered a poor prognosis lymphoma; a retrospective analysis more recently published found a trend in improved overall survival, with an estimated survival of 53% at 4-years in the 2013-2016 cohort, compared to 38.1% in the 2003-2006 group (10).

Differently from PCL, secondary cardiac involvement from lymphoma is a relatively frequent occurrence, reported in up to 25% of patients with nodal disease (11); the incidence is probably underestimated, due to a percentage of asymptomatic cases. Most frequently secondary cardiac involvement occurs in patients with DLBCL (2–5) while it is more rare in patients suffering from CLL/SLL, follicular lymphoma and T-cell lymphoma (12, 13) and extremely rare (14) in Hodgkin lymphoma. In secondary cardiac lymphoma (SCL) symptoms are non-specific, frequently attributed to other causes and concomitant conditions; therefore in a proportion of patients cardiac involvement is not even sought. Clinical presentation is similar to that of PCL, with symptoms attributable to heart failure or rhythm alterations in the majority of cases (5).

Due to the rarity of this condition, several case reports and few reviews have been reported so far. Here we describe three cases of cardiac involvement from lymphoma that were observed at our institution in these last years. These cases show how heterogeneous can be the presentation and the clinical behavior of cardiac lymphoma and highlight some issues that clinicians may face in the management of this disease.

## Cases Description Case Report 1

A 72-years-old Caucasian man presented on March 2019 with worsening dyspnea. Physical examination showed mild bibasal reduction of vesicular murmur, slight jugular turgor, mild hepatomegaly, rhythmic cardiac activity, and normal blood pressure. Echocardiography revealed a mass in the right atrium. The total body CT scan confirmed the mass in the right atrium and superior vena cava, and showed conspicuous bibasal pleural effusion. A few days later the patient developed atrial fibrillation, which was cardioverted with amiodarone. Echocardiography showed a slight increase in pericardial effusion with signs of hemodynamic compromise. The patient underwent surgical debulking. Histology showed the presence of widespread proliferation of large-sized lymphocytes positive for CD20, CD5, CD10, Bcl-2, Bcl-6, PAX5, MUM1, high Ki67 (80%), consistent with DLBCL CD5 positive, GCB-type according to Hans algorithm. IPI score resulted low-intermediate (age, increased LDH).

The patient was then referred to our Department and started on chemoimmunotherapy according to the R- COMP scheme (Rituximab, Cyclophosphamide, non-pegylated Liposomal Doxorubicin, Vincristine, Prednisone) for six courses every 21 days, followed by two administrations of Rituximab alone. Liposomal Doxorubicin was chosen due its lower cardiac toxicity. The echocardiography performed after 4 courses of therapy showed normal size, kinetics and pump function of the left ventricle and normal atria. The CT-PET scan performed after the 6^th^ R-COMP course showed a complete metabolic response.

At the check-up prior to the eighth Rituximab course, the patient reported pain and disorders of the visual acuity in the left eye. A brain MRI was consistent with central nervous system (CNS) lymphoma localization involving the lenticular nucleus, internal and external capsule of the left hemisphere and the left optic nerve. Lumbar puncture showed the presence of pathological monoclonal B-lymphocytes, which confirmed CNS relapse. Cardiac MRI revealed no elements of disease recurrence. The patient was then treated with a single course of dose-adapted Methotrexate, Cytarabine, Thiotepa, Rituximab (MATRIX chemotherapy) with poor tolerance and development of acute renal failure. For this reason the patient was treated with CNS radiotherapy followed by 5 cycles of temozolomide, dexamethasone and intra-thecal methylprednisolone, cytarabine, methotrexate achieving partial response. The patients died on April 2020 because of disease progression.

### Case Report 2

**Figure 1:**
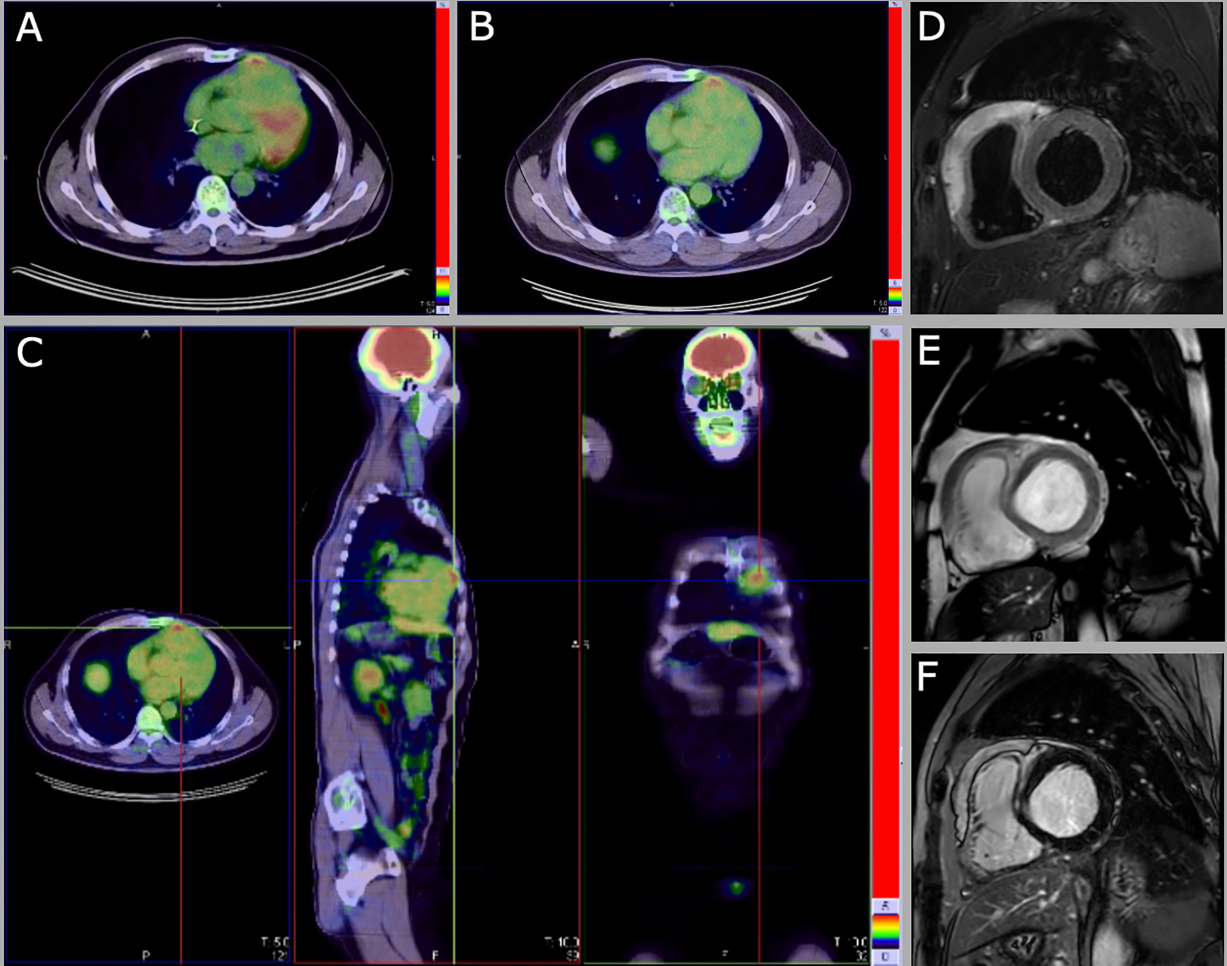
CT-PET abnormal 18-FDG uptake on right ventricle thickening 3 months (SUV 4.9, DS 4) **(A)** and 18 months (SUV 5 – DS 4) **(B, C)** after ASCT. Cardiac MRI images of June 2019 (from **D** to **F**) show the persistence of the right ventricle anterior wall thickening (14x56 mm). **(D)** Short tau inversion recovery (STIR) T2 black blood image in short axis on the cardiac base. **(E)** Steady-state free precession (SSFP) balance image with T2/T1 weighting in short axis on the cardiac base. **(F)** IR TSET1 image for evaluation of myocardial late enhancement in short axis on the cardiac base.

A 56-years-old Chinese man was referred on December 2017 to our Center with a diagnosis of primary cardiac DLBCL, IPI score low (0), treated with 6 cycles R-CHOP in China from January to May 2017. A restaging CT-PET performed in October 2017 showed an abnormal 18-FDG uptake on the right ventricle (SUV 4.7) and absence of any other lymphoma localization; an echocardiography and a cardiac MRI revealed a mass of 3 x 5 cm infiltrating the anterior wall of the right ventricle. An endomyocardial biopsy was not performed because considered too risky. The patient was then started to second-line chemotherapy R-ICE (rituximab, etoposide, carboplatin, ifosfamide) and consolidation with autologous stem cell transplantation (ASCT; FEAM conditioning). Cardiac MRI performed before the ASCT showed only a slight reduction of the right ventricle thickening (11 mm vs 13 mm). Echocardiography performed shortly after ASCT showed the persistence of a hyperechoic mass infiltrating the anterior wall of the right ventricle (46x10 mm). The possibility of a myocardial biopsy to confirm the relapse was discussed with the cardiologists, but due to the risks related to the procedure, and the substantial asymptomaticity of the patient, he was kept under close observation. Three months after ASCT, a cardiac MRI was repeated, which resulted unchanged; a CT-PET was performed, showing abnormal 18-FDG uptake on right ventricle thickening (SUV 4.9 – Deauville score (DS) 4) – **Figure 1A**. Watch and wait approach was pursued, due to the stability of the radiological findings and the asymptomaticity of the patient. Cardiac MRIs performed on October 2018, June 2019 and November 2019 showed a substantial stability of the picture, with the persistence of the right ventricle anterior wall thickening – **Figures 1D–F**. The CT-PET performed in June 2019 confirmed the abnormal 18-FDG uptake on right ventricle thickening (SUV 5 – DS 4) – **Figures 1B, C**. Echocardiography showed substantial stability. The patient is now 2 years off-therapy, with both clinical and radiological stability, in clinical follow-up. The “watch and wait” strategy, although eventually correct, raised many concerns and fears in the patient, and generated anxiety related to the impossibility to ascertain the disease status.

### Case Report 3

**Figure 2:**
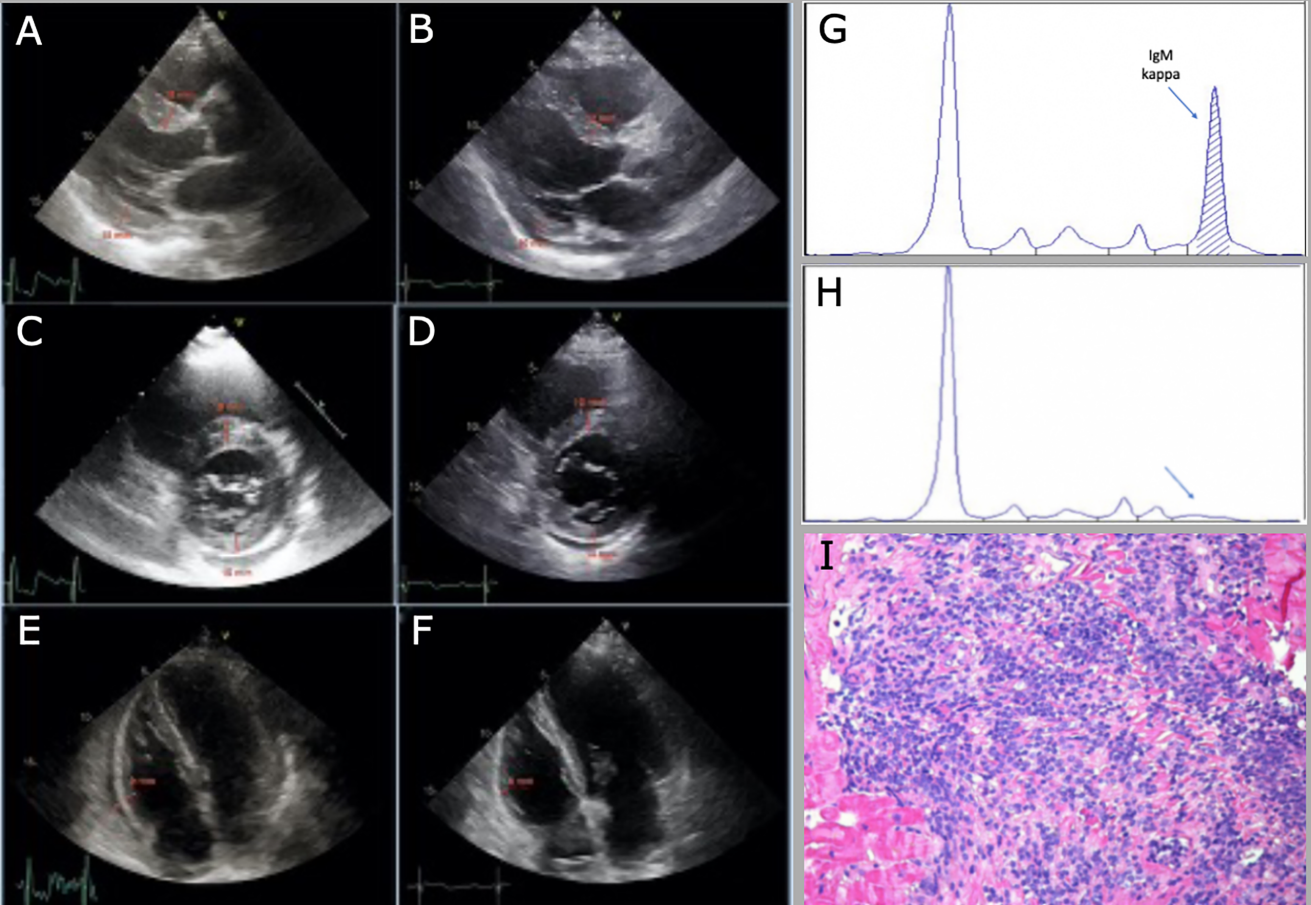
Decrease in cardiac wall thickness after chemotherapy at echocardiographic evaluation (from **A** to **F**). **(A, C)** interventricular septum (IVS) and posterior wall (PW) thickness, 18 mm and 15 mm respectively, from parasternal long-axis and short-axis view, before chemotherapy. **(B, D)** IVS and PW thickness, 12 mm and 10 mm, respectively, from parasternal long-axis and short-axis view, after chemotherapy. **(E, F)** right ventricular lateral wall (RV-LW) thickness, 9 mm and 6 mm, respectively, from 4 chamber view; of note, pericardial effusion decreases from 18 mm to 6 mm after chemotherapy. **(G)** monoclonal IgM/K before starting treatment. **(H)** no more detectable monoclonal paraprotein after treatment. **(I)** histologic sample of endomyocardial biopsy, hematoxylin-eosin staining.

A 70-years-old Caucasian man presented on May 2019 to the Cardiology department with new-onset, worsening dyspnea (NYHA II) and episodes of tachycardia with spontaneous resolution. Physical examination showed tachycardia (110 beats/min), mild hypotension (115/75 mmHg), systolic murmur grade 2/6; no clinical findings suggestive of heart failure, no lymphadenopathies nor organomegalies. The ECG showed sinus tachycardia, first degree atrioventricular block, left anterior hemiblock, minor right bundle branch block. Echocardiography revealed biventricular hypertrophy, mild bi-atrial dilatation, interventricular septum thickening (18 mm), mild pericardial effusion (18 mm), with normal kinetics and pump function (ejection fraction left ventricle 62%) - **Figures 2A, C, E**. Chest x-ray showed hilar enlargement, without pleural effusion. Blood tests showed mild normocytic anemia (Hemoglobin 128 g/L), normal platelet count, white blood cell count and differential, increased ESR (87 mm/h; ULN 35 mm/h), normal LDH, renal and liver function, BNP and Troponin I. An IgM/K paraprotein of 20 g/L was found on serum protein electrophoresis, with decreased IgG and IgA levels (5.4 g/L and 0.48 g/L respectively), and an altered serum free light chain ratio (serum free light chain kappa 411 mg/L, lambda 6.72, kappa/lambda ratio 61.21) – **Figure 2G**. Serologies for HBV and HCV were negative. In the suspicion of cardiac amyloidosis, a cardiac scintigraphy was performed, which resulted negative for cardiac transthyretin- related amyloidosis (score 1).

The patient was then referred to our Department; a bone marrow trephine showed a massive infiltration of small-sized lymphocytes positive for CD20 and Bcl-2, negative for CD5, CD23, CD138, CD10, Cyclin D1, SOX11, Ki67 was low (3-5%), consistent with infiltration from marginal zone lymphoma (MZL); Congo red- staining for amyloidosis was negative; the number of plasma cells was normal. For a strong suspicion of AL amyloidosis, abdominal fat pad and salivary glands biopsy were performed: both resulted negative for Congo red-staining. Cardiac MRI showed concentric hypertrophy of the left ventricle, without signs of cardiac amyloidosis or Fabry disease, and persistence of pericardial effusion (max 15 mm). Clinically the patient developed several episodes of atrial flutter/atrial fibrillation with rapid ventricular response, which required a biventricular pace-maker implantation. An endomyocardial biopsy showed massive cardiac infiltration by B-lymphocytes from MZL, with no signs of amyloidosis – **Figure 2I**. A total-body CT-scan showed mild supra and sub-diaphragmatic lymphadenopathies (max 29 mm); mild splenomegaly (16 cm). 18-FGD-PET didn’t show any increased uptake in lymphadenopathies nor in the myocardium. Immunophenotype on peripheral blood revealed a tiny population of clonal CD19+CD20+ kappa B cells (0.12% of total WBC). MALT IPI score was high (2: age, stage).

The patient was started on Bendamustine – Rituximab, for 6 courses every 28 days. After 4 courses of therapy the echocardiography displayed a substantial stability of the picture, with persistence of mild bi- atrial dilatation and pericardial effusion (max 10 mm). After six courses of therapy, bone marrow trephine was negative for lymphoma, and flow cytometry on bone marrow aspirate didn’t find clonal B cells. On serum protein electrophoresis the monoclonal paraprotein was not detectable – **Figure 2H**. CT-scan showed a radiologic complete response according to Lugano criteria. Echocardiography showed a reduction of interventricular septum thickness (12 mm), normal function of left ventricle (ejection fraction 72%), bi- atrial mild dilatation and reduction of pericardial effusion (6mm) - **Figures 2B, D, F**. Cardiac MRI was not performed due to the presence of pace-maker, which shields a significant part of the myocardium. Due to the clinical, laboratory and radiologic response and the negativity of bone marrow examination, and considering the risks associated with the procedure, endomyocardial biopsy was not repeated.

## Discussion

We reported three cases of cardiac involvement from lymphoma: two PCL and a unique case of secondary cardiac involvement from MZL.

Due to the rarity of this condition, isolated case reports and a few reviews have been published in literature so far, which include a population collected in a wide period of time and heterogeneously managed. Petrich et al (2) collected 197 cases of cardiac involvement from lymphoma (1949-2009), of which 68% met strict criteria for PCL. Median reported OS was 12 months. More recently, Chen et al. published a collection of cardiac lymphoma case reports from 2009 to 2019, including 101 PCLs (15). Voigt et al (4) collected 616 cases of cardiac hematological malignancies, including 558 patients with cardiac involvement from lymphoma, which was DLBCL in 77% of cases. Our two PCL confirm what previously reported: male predominance, onset with cardiac symptoms, involvement of the right chambers and DLBCL histology. Both patients were treated with first-line R-CHOP.

*Case report 1* points out the issue of CNS prophylaxis in patients with PCL. In patients with DLBCL, CNS-IPI (16) has been used to identify high-risk patients, however some disease characteristics, such as involvement of specific sites (e.g.: testicular, breast), remain a risk factor for CNS involvement and requires CNS prophylaxis. Our patient had a CNS-IPI score 2 (intermediate-risk: age > 60 years, increased LDH) before treatment and experienced CNS progression very shortly after the end of R-CHOP. We didn’t find any specific recommendation in literature on this topic and in the two large literature reviews previously reported no CNS progression was described (2, 4). A few case reports (17–21) described isolated parenchymal CNS relapse in patients with PCL: in most cases, similarly to what happened in our patient, relapsed occurred shortly after the completion of chemotherapy (within two months) in responders. Only one patient had received CNS prophylaxis with intermediate-dose (1.5 g/m^2^) methotrexate, administered every two weeks for four doses (20). A proportion of these patients responded to second-line therapy, but long-term follow-up is missing.

Based on these previous experiences and literature reviews, it seems that CNS relapse in PCL is a rare but possible occurrence. Due to its particular solitary extranodal location, CNS-IPI is probably not the best tool to assess the risk of CNS involvement in PCL. According to our and previous experiences in literature, CNS evaluation at baseline and CNS prophylaxis with methotrexate should be considered early in DLBCL fit patients, concomitantly with R-CHOP or as a single agent after the first three or four R-CHOP courses; further studies, however, are needed in order to better clarify this issue.

*Case report 2* highlights the pitfalls of radiologic assessment during follow-up that, as it can be expected, generated profound anxiety and uncertainties in the patient and physicians. Indeed, multiple cardiac MRIs, 18-FDG-PET/CTs and echocardiography showed the persistence of cardiac involvement in the two years follow-up after ASCT, which contrasted with the wellbeing and clinical stability of the patient. Despite the radiological findings and considering the aggressive histology and the cardiac location, we are rather confident that the patient does not have an active disease, which would have become manifest meanwhile. The less reliable test, in this case, was 18-FDG-PET/CT, which continued to detect a pathological 18-FDG uptake, remaining positive according to Lugano criteria (Deauville score 4). Being aggressive lymphomas, PCLs are usually 18-FDG avid tumors (22), however, the utility of FDG-PET/CT alone in the diagnosis and follow-up of cardiac involvement is controversial, due to the physiological uptake of the heart and the low anatomic resolution of FDG-PET (3, 23).

To our knowledge *case report 3* describes for the first time a secondary cardiac involvement by MZL. Gordon et al. (5) collected from literature 94 cases of cardiac non-Hodgkin lymphoma, including 43 patients with secondary cardiac involvement. Most of these patients were males (67%), in one third of cases aged > 60 years. The most common histologic subtype was DLBCL (42%), followed by T-cell lymphoma (33%),

Burkitt lymphoma (9%), CLL/SLL and follicular lymphoma (4% each). No cases of secondary involvement of the heart by MZL were reported. MZLs are a group of indolent B-cell lymphomas that derive from memory B lymphocytes of marginal zone origin and comprise extranodal MZL of mucosa-associated lymphoid tissue (also known as MALT lymphoma), splenic MZL and nodal MZL. They can arise at any extranodal site, more frequently in the contest of chronic antigen stimulation (24). In our patient, the clinical picture was dominated by cardiac symptoms, in particular rhythm abnormalities, which led to further investigations. The first hypothesis to explain echocardiography and ECG results was cardiac amyloidosis, AL-type due to the presence of monoclonal paraprotein, however some findings did not match with this suspicion: BNP and troponin were negative, cardiac scintigraphy and cardiac MRI were not suggestive for cardiac amyloidosis and Congo red staining resulted negative in bone marrow, fat pad and salivary gland biopsies. Furthermore, AL amyloidosis secondary to lymphoma has been reported in 2-4% of cases (25), while no cases of cardiac amyloidosis secondary to MZL have been published so far. Once cardiac infiltration by lymphoma was found, the patient started chemo-immunotherapy with Bendamustine and Rituximab, as per our clinical practice for indolent lymphomas, and in order to avoid anthracycline-related cardiotoxic effects. A further issue that we experienced with this patient was the evaluation of the response at the end of treatment, since 18-FDG-PET, which was negative at baseline, resulted negative at restaging as well, cardiac-MRI was not reliable due to the pacemaker implant, and the only radiological test on which we could rely was the echocardiography. The only test which could provide a response would have been the EMB, but due to the risks associated with the procedure, we preferred to avoid it, considering that all the remaining parameters of activity of disease showed a response to therapy.

Through these experiences, we observed that sometimes the complicated medical management, especially in rare disease where guidelines are lacking, could impact the patient’s wellbeing.

In conclusion, primary or secondary cardiac lymphoma are a rare well described conditions that require careful inspection in patients with lymphoma and cardiologic signs or symptoms. Given the heterogenous pattern of presentation this condition often requires individualized management and a multidisciplinary approach.

## Data Availability Statement

The original contributions presented in the study are included in the article, further inquiries can be directed to the corresponding author.

## Ethics Statement

Ethical review and approval was not required for the study on human participants in accordance with the local legislation and institutional requirements. The patients/participants provided their written informed consent to participate in this study. Informed consent was obtained from the relevant individuals, and next of kin, for the publication of any potentially identifiable images or data included in this article.

## Author Contributions

EL: her contribution was to design the project, collect the patients’ clinical history patients, review the literature, and write the paper. MM: his contribution was to outline and integrate the cardiological management of patients in the paper. MB: his contribution was to collect the patients’ clinical history and write the paper. AP: his contribution was to outline and integrate the cardiological management of patients in the paper. GS: his contribution was to outline and integrate the cardiological management of patients in the paper. LP: his contribution was to outline the radiological management of patients with particular reference to case report 2. MR: his contribution was to outline the nuclear medicine management of patients with particular reference to case report 2. AR: his contribution was to characterize histological samples with particular reference to case report 3. RB: her contribution was to characterize histological samples with particular reference to case report 3. LB: her contribution was to collect the patients’ clinical history and write the paper. FZ: he directed the project and also helped to design and write the paper. All authors contributed to the article and approved the submitted version.

## Conflict of Interest

The authors declare that the research was conducted in the absence of any commercial or financial relationships that could be construed as a potential conflict of interest.
